# Thyroid Nodule Detection and Region Estimation in Ultrasound Images: A Comparison between Physicians and an Automated Decision Support System Approach

**DOI:** 10.3390/diagnostics13182873

**Published:** 2023-09-07

**Authors:** Elmer Jeto Gomes Ataide, Mathews S. Jabaraj, Simone Schenke, Manuela Petersen, Sarvar Haghghi, Jan Wuestemann, Alfredo Illanes, Michael Friebe, Michael C. Kreissl

**Affiliations:** 1 Division of Nuclear Medicine, Department of Radiology and Nuclear Medicine, University Hospital Magdeburg, 39120 Magdeburg, Germany; simone.schenke@klinikum-bayreuth.de (S.S.); michael.kreissl@med.ovgu.de (M.C.K.); 2 Otto-von-Guericke University Magdeburg, 39106 Magdeburg, Germany; 3 Department of Nuclear Medicine, Klinikum Bayreuth, 95445 Bayreuth, Germany; 4 Department of General, Visceral, Vascular and Transplant Surgery, University Hospital Magdeburg, 39120 Magdeburg, Germany; 5 Department of Nuclear Medicine, University Hospital Frankfurt, 60590 Frankfurt, Germany; 6 Surag Medical GmbH, 39118 Magdeburg, Germany; 7 Department of Biocybernetics and Biomedical Engineering, AGH University of Science and Technology, 30-059 Krakow, Poland; 8 Center for Innovation, Business Development and Entrepreneurship (CIBE), FOM University of Applied Science, 45127 Essen, Germany; 9 STIMULATE Research Campus, 39106 Magdeburg, Germany; 10 Center for Advanced Medical Engineering (CAME), Otto-von-Guericke University Magdeburg, 39106 Magdeburg, Germany

**Keywords:** ultrasound, thyroid nodules, detection, classification, subjectivity, observer variability, computer aided diagnosis

## Abstract

Background: Thyroid nodules are very common. In most cases, they are benign, but they can be malignant in a low percentage of cases. The accurate assessment of these nodules is critical to choosing the next diagnostic steps and potential treatment. Ultrasound (US) imaging, the primary modality for assessing these nodules, can lack objectivity due to varying expertise among physicians. This leads to observer variability, potentially affecting patient outcomes. Purpose: This study aims to assess the potential of a Decision Support System (DSS) in reducing these variabilities for thyroid nodule detection and region estimation using US images, particularly in lesser experienced physicians. Methods: Three physicians with varying levels of experience evaluated thyroid nodules on US images, focusing on nodule detection and estimating cystic and solid regions. The outcomes were compared to those obtained from a DSS for comparison. Metrics such as classification match percentage and variance percentage were used to quantify differences. Results: Notable disparities exist between physician evaluations and the DSS assessments: the overall classification match percentage was just 19.2%. Individually, Physicians 1, 2, and 3 had match percentages of 57.6%, 42.3%, and 46.1% with the DSS, respectively. Variances in assessments highlight the subjectivity and observer variability based on physician experience levels. Conclusions: The evident variability among physician evaluations underscores the need for supplementary decision-making tools. Given its consistency, the CAD offers potential as a reliable “second opinion” tool, minimizing human-induced variabilities in the critical diagnostic process of thyroid nodules using US images. Future integration of such systems could bolster diagnostic precision and improve patient outcomes.

## 1. Introduction

High-resolution US is the most important imaging modality for the characterization of thyroid nodules (TNs). It is an easy-to-apply and available imaging modality that does not involve ionizing radiation and is non-invasive. In addition to these benefits, it is able to provide high-resolution soft tissue imaging [1]. The evaluation of TNs using US imaging is based on visual characteristics, which are documented during the examination. For this purpose, the Thyroid Imaging Reporting and Data System (TIRADS) approach is used for the risk stratification and classification of benign and malignant nodules. An example of such a classification system is by Kwak et al. (Kwak-TIRADS) which relies on the following five US features being suspicious for malignancy: solid composition, hypoechogenicity, irregular/microlobulated margin, micro-calcification, and a taller-than-wide shape [2,3]. Leveraging this classification system is dependent on the examining physician’s experience with the imaging modality and the system itself. Of course, physicians having more experience are likely to perform a more precise classification as opposed to those with less experience [4]. Hence, the process of observation is riddled with subjectivity and observer variability. Upon further investigation, the observer variability has a trickle-down effect on other aspects of the TN and risk-stratification process. Park et al., in their assessment of sonographic TNs, found that the agreement among examining physicians was the lowest in the context of margin detection and echotexture [5]. Similarly, Lee et al. found that observer variability between two physicians diagnosing TNs using US images varied with their experience [6]. In certain cases, high subjectivity can lead to an overestimation or underestimation of the risk for malignancy of nodules [7]. Seifert et al. in their study discussed the subjectivity in TN classification among different physicians using various TIRADS classification methodologies [8].

An efficient approach to reduce subjectivity is to provide physicians with a decision support system (DSS) that uses quantitative computation and aids the overall classification process. A DSS is beneficial as it often takes into consideration features that are not visible right away to the physicians for the segmentation (detection), risk stratification, and classification of TNs on US [9]. DSSs are developed using several methodologies, of which the two most recent ones are Machine Learning (ML) and Deep Learning (DL).

### 1.1. ML and Hand-Crafted Feature Extraction for DSS

To use ML for the development of a DSS, it needs to be coupled with features that provide the ML algorithm with characteristics of a structure that helps classify it. The most commonly used features are hand-crafted. Hand-crafted features refer to features purposefully extracted and custom-designed to meet the requirements of the problem statement as opposed to features extracted at random, as in the case of DL. Savelonas et al. in their work used Radon domain features that characterize thyroid tissue textures based on the differences in directional patterns [10]. Local binary patterns are also an example of hand-crafted features that can be used for the detection of thyroid nodules and have resulted in increased accuracy [11]. Similarly, Keramidas also put forth the use of Fuzzy Local Binary Pattern features for the further improvement of thyroid nodule detection based on their textures [12]. Another example of hand-crafted features for the segmentation of thyroid nodules on US images is the gray-level co-occurrence matrix that enables the extraction of second-order statistical features [13]. Using hand-crafted features in combination with ML, a TN detection system was developed for the analysis of 2D thyroid US images and videos [14]. Chang et al. employed decision trees as an ML approach together with 41 hand-crafted features derived from the co-occurrence matrix, statistics, and gray-level run matrix [15]. Novel ways of deriving hand-crafted features were observed through the work presented in [16], where texture-based features were extracted by using parametrical modeling by converting US image patches into signals. These features coupled with ML provided a robust classification of thyroid tissue textures.

### 1.2. DL for DSS

Chi et al. carried out the classification of US TNs using a fine-tuned deep convolutional neural network (pre-trained GoogleNet model), which resulted in a classification accuracy of 98.3% and 96.3% on two separate databases [17]. A deep learning network, RS-Net or risk stratification network was developed by Bai et al. and achieved an accurate risk stratification of up to 88.0% when ACR TI-RADS was used [18]. ThyNet, another deep learning model, was developed to differentiate between benign and malignant tumors in order to improve diagnostic performance. ThyNet accurately diagnosed thyroid nodules in up to 92.2% of cases, significantly surpassing the accuracy of the reading physicians of 83.9% [19]. When considering the segmentation of TNs, Ma et al. developed a deep convolutional neural network for 2D thyroid US images that resulted in an overlap of 86.83% with the ground truth [20]. Technologies such as cascading convolutional neural networks also aid in the detection of thyroid nodules with an overlap of 87.00% with the ground truth [21]. Kumar et al. also developed a multi-output convolutional neural network to segment TNs as well as the cystic regions in US images and achieved an overlap of 76.00% with the ground truth [22].

The aforementioned literature treads along a roadmap that begins with exhibiting the presence of observer variability in the detection, risk stratification, and classification of TNs and ends with the current technological advances using ML and DL. It shows that the development of DSSs for US TN diagnosis is rapidly increasing. With each iteration, DSSs are closer to matching the performance of an experienced physician. This tells us that DSSs have the ability to provide support to the physician during the diagnostic process [23]. A DSS is meant to support the physician’s decision without being influenced by external bias or human subjectivity.

In this study, we aim to add to the domain by first presenting technological advances of our own in the form of a DSS and further evaluating them with the help of practicing physicians by comparing the two in an attempt to show that the DSS can be closer in performance to an experienced physician. We used three physicians with varying levels of experience and compared their performance against TN detection and region estimation solutions that were developed. Region estimation is a vital component of the classification process as nodules that possess a larger solid portion have a higher risk of being malignant, whereas those with a larger cystic portion often prove to be benign [4]. Also, estimating the cystic percentage of a nodule can be helpful in determining the truly active volume of an autonomously functioning nodule in the context of planning for radioiodine treatment. All in all, it is helpful for the physician to have an objective region estimation approach for an efficient risk stratification of TNs. The DSS employs a Deep Neural Network to detect the TN followed by the employment of hand-crafted features to estimate the solid and cystic regions within the nodule.

## 2. Materials and Methods

### 2.1. Patients and US Data Collection

The data used in this study were collected prospectively after obtaining ethical approval (RAD362-16/19) at the Otto-von-Guericke University Hospital, Magdeburg. Prior to the data collection, informed consent was obtained from each patient. A total of 20 patients were enrolled. Patients having TNs between ages 18 and 80 were included in the study; patients who underwent radioiodine therapy and those with known Hashimoto’s Thyroiditis were excluded. Image data were acquired using a current US device (Logiq S8, GE Healthcare, Madison, WI, USA) equipped with a linear probe (ML6-15-D linear probe) with a base frequency of 12 MHz, as 3D US datasets using an electromagnetic tracking system. This probe was selected as it was most frequently used during routine processes for thyroid image acquisition. The datasets acquired were of the thyroid gland; the region of the nodule was recorded in two planes (transverse and longitudinal). The data were collected in the form of tracked US videos. Each of the 20 patients had an average of 3 nodules (1–5 nodules per patient resulting in a total of 56 nodules found in total in 20 patients). For the development of the DSS, independent frames consisting of different views (longitudinal and transverse) from each of the nodules were extracted and further augmented using data augmentation techniques. A total of 1011 final images were obtained. The data were collected by three highly trained nuclear medicine physicians experienced in US imaging. Since the data were collected prospectively in a clinical setting, the appropriate clinical protocols were followed to ensure data quality.

### 2.2. Experimental Setup

Nodules from 20 patients were assessed; they were selected based on how different the nodules were in terms of texture and size, i.e., each nodule selected had a combination of two different regions (solid and cystic). The images were analyzed independently for compositional anomalies by the physicians as well as by the DSS-based approach.

#### 2.2.1. Analysis by Physicians

The images were analyzed independently by three nuclear medicine physicians who have varying degrees of experience at the Division of Nuclear Medicine, Department of Radiology and Nuclear Medicine, Otto-von-Guericke University Magdeburg. The reading process was carried out as follows. Each of the participating physicians first segmented the thyroid nodules. The average of the segmentations was taken and used as the ground truth for the DSS. To further elaborate on this process, each ground truth annotation was used to generate a binary mask of the nodule. The area of binary masks (in mm^2^) from three physicians was combined to obtain an average area (mm^2^). This was then converted back into a binary mask and used as the final ground truth. This averaging technique was also applied to generate the ground truth for solid and cystic regions. Once both ground truths were generated (full nodule and solid and cystic regions), the full nodule data were used in the DL algorithm of the DSS. Additionally, the percentage of cystic regions of the nodules was estimated by the physicians. Physician 1 has 8 years of experience classifying thyroids, while Physicians 2 and 3 each have 2 years of experience classifying TNs by ultrasound. Kwak-TIRADS was used [4].

#### 2.2.2. Computational Analysis

Technological solutions using DL and ML were developed for detecting TNs in US images and quantifying their solid and cystic regions to form part of a DSS. Each of these are highlighted below.

The detection of the nodules was first investigated through a comparison of state-of-the-art semantic segmentation models. Through this investigation, it was determined that the ResUNet architecture performed the best in terms of accuracy of segmenting and thereby detecting thyroid nodules in US images [24]. The better performance of the ResUNet architecture compared to the other selected architectures in [24] can be attributed to the presence of residual blocks, which enables consistent training of the network as the depth of the network increases.

During the solid and cystic region estimation phase, the segmented nodules along with their clustered regions were divided into texture patches of size 20 × 20 pixels or 5.296 × 5.296 mm. These patches were converted into four signals using the method described in detail in [25]. The signals were then decomposed and separated into three distinct frequency bands before employing auto-regressive modeling to extract features based on textural differences found in the cystic and solid regions. A total of 36 features were extracted, labeled, and then fed into ML models that classified the regions within the thyroid nodule. The best-performing model (Random Forest Classifier) was selected and the classification outputs were used to estimate the percentage of each region [26].

### 2.3. Statistical Analysis

A chi-squared statistical test for significance was performed to determine the variance between physicians and the DSS approach. The test was performed using GraphPad Prism v8.4.2. (by Dotmatics—Boston, MA, USA).

## 3. Results

### 3.1. Nodule Detection by Physicians and DSS

**Figure 1:**
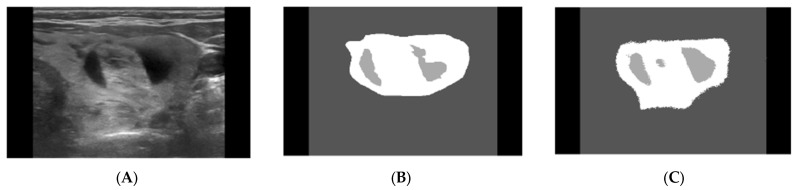
Example of segmentation (nodule detection) results obtained using the ResUNet model. (**A**) Original image, (**B**) average of Physicians 1, 2, 3 detection, and (**C**) ResUNet segmentation [24].

For this step, an average of the nodule detections for each of the physicians was taken and compared against the nodules as detected by the ResUNet approach. An example of this can be seen in Figure 1. Figure 1A depicts the original thyroid nodule US image, Figure 1B depicts the average from three physicians for the ground truth of the nodule, and Figure 1C is the nodule as detected by the DSS approach.

**Figure 2:**
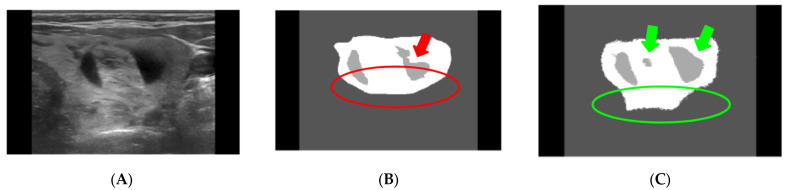
Example of segmentation (nodule detection) results obtained using the ResUNet model. (**A**) Original image, (**B**) average of Physicians 1, 2, 3, and (**C**) ResUNet segmentation. The red and green indications are differences seen in the segmentation [24].

Variability in the nodule detection process is seen due to US images being dappled with speckle noise and artifacts, making detection a challenging task. Figure 2B depicts the average for the ground truth of the nodule from the three physicians. Figure 2C is the nodule as detected by the DSS approach. Both images adequately detect the thyroid nodule in the US images. However, the DSS-based approach was able to detect regions of the nodule that were missed during the assessment by the physicians. The differences in the segmentation can be seen in the regions indicated by the colors (red and green) in Figure 2B,C.

### 3.2. Comparison between DSS Region Estimation and Physician Region Estimation

Table 1 shows the percentage of matches between all the physicians and the DSS and individual physicians and DSS for the overall classification of the thyroid nodules. The classification match percentage for each individual physician vs. DSS is calculated by comparing the number of cases classified as the same by the physician and the DSS. For all physicians vs. DSS, the classification match percentage is calculated by first averaging the number of cases classified as the same by each physician and then comparing the average to the DSS classifications. It can be seen that the percentage of all physicians and the DSS resulting in the same classification of the nodules is only 19.2% of the total observations made. Physician 2 and the DSS had the same classification for 42.3% of the observations. Physician 3 and the DSS resulted in equal classification of the nodules 46.15% of the time. Physician 1 and the DSS yielded the highest match percentage for the classification, which was 57.6%.

Of the 1011 total images analyzed, 26 images were selected at random from the 20 cases. Only a single image slice from a TN was chosen from all the images extracted. The slice chosen was located in the center of the nodule so that it contained the largest vertical and horizontal axes. These were used for the presentation of the results as follows. Table 2 shows the comparison in observations for each physician and the DSS with respect to determining the region estimations within a nodule. Only the cystic percentage estimations have been shown, as the remainder is assumed to be solid in nature. Each of the physicians estimated different percentages for each region. This can be attributed to the difference in perception of the quantities of these two regions within the nodule. This reflects the variability between the observers for each observation. Additionally, the DSS estimates the regions differently while also leaning more towards the physician with a higher level of expertise in a majority of the observations.

Table 3 shows the variance percentage for each of the selected observations between each of the physicians and the DSS for cystic region estimation. Variance percentage is defined as the difference between Observer 1 and Observer 2 divided by Observer 1 and multiplied by a factor of 100. In this case, Observer 1 represents each of the physicians in each case (1, 2, or 3), while Observer 2 is the DSS and remains constant throughout. Variance percentage can be interpreted in different ways. A positive variance percentage indicates a positive deviation between the two observers. A null value (NA) is returned when there is an invalid deviation, as seen in the case of nodule 6 for Physician 1. A negative variance percentage is indicative of a deviation between the observers when Observer 2 estimates a region that is greater in value when compared to Observer 1. Negative variance percentages are not a bad occurrence in terms of nodule region estimation, as it is indicative of a difference in estimation criteria that is considered by the DSS as opposed to the physician. In Table 3, the most negative variance percentage occurrences can be seen between the DSS and Physician 3. The lowest occurrences are seen in the case of the DSS and Physician 1 among the examples considered. This is attributed to the difference in experience levels of the physician and can be further associated with the presence of higher variability levels in lower experience scenarios. The higher negative variance percentage occurrences can also be reduced by more use of the DSS by physicians with lower experience levels.

Through the region estimation phase, it can be seen that there was a difference between the observations made solely by the physicians. The region estimation and subsequent quantification of solid and cystic components is a vital step in the classification of TNs using US images.

## 4. Discussion

Through this study, it is understood that there are several factors that influence the classification of TNs using US images. The experience of the physician is a main factor. The DSS is designed to reduce and limit such subjectivity. These systems identify patterns and features in images that cannot be seen by visual inspection of the image. These unseen features are extracted automatically by Deep Learning algorithms such as in the case of [24] or manually determined as in the case of [27]. These systems are mathematically proven and function with minimum human intervention during the classification process. Another study group set up their DSS to provide a possible diagnosis in two formats (Korean Society of Thyroid Radiology (K)-TIRADS and the American Thyroid Association (ATA)-TIRADS classifications), as well as results (possibly benign or possibly malignant). They showed the diagnostic sensitivity, specificity, positive predictive value, negative predictive value, and accuracy of the DSS for thyroid cancer, which were 97.6%, 21.6%, 42.0%, 93.9%, and 49.6%, respectively, for K-TIRADS; 94.6%, 29.6%, 43.9%, 90.4%, and 53.5%, respectively, for ATA-TIRADS; and 81.4%, 81.9%, 72.3%, 88.3%, and 81.7%, respectively, for dichotomous outcomes. The sensitivities of the computer-aided diagnostic system did not differ significantly from those of the radiologist (all *p* > 0.05); the specificities and accuracies were significantly lower than those of the radiologist (all *p* < 0.001) [28]. Zheng et al. evaluated the diagnostic performance of different US sections of 221 TN using DSS based on artificial intelligence (AI-CADS) in predicting thyroid malignancy. Patients with preoperative US data and postoperative pathological results were enrolled and divided into two groups: lower-risk group (ACR TI-RADS 1, 2 and 3) and higher-risk group (ACR TI-RADS 4 and 5). The malignant risk scores of nodules were obtained from longitudinal and transverse sections using AI-CADS. The diagnostic performance of AI-CADS and the consistency of each US characteristic were evaluated between these sections. The diagnostic performance of DSS based on artificial intelligence (AI-CADS) in longitudinal and transverse ultrasonic views for differentiating TNs was different, being higher in the transverse section. It was more dependent on the section for the AI-CADS diagnosis of suspected malignant TNs [29].

In any case, it is poignant to understand that DSS, when used, is not meant to replace physicians but rather acts as a support system to validate their decisions or provide them with a “second opinion” to facilitate improved decision making. For instance, though the estimation of solid and cystic regions may seem straightforward, it does introduce observer variability. This could prove to be harmful to the patient during risk stratification and maybe even lead to inaccurate treatment planning. The estimation of solid and cystic regions using the DSS approach can be used to curb the observer variability by near-accurate quantification and provide a sort of scoring system for the nodule. This is similar to the scoring system used by the ACR TIRADS [30], which should be adopted for a DSS approach as well. This in turn would aid in the overall risk stratification process in a much more comprehensive way. It is vital to note that the estimation of regions needs to be made with respect to the entire nodule itself. This means that the volume of the nodule also needs to be taken into consideration if the TIRADS classification is to be performed efficiently by a DSS. Keeping this in mind, it is safe to say that this process will be computationally very demanding if it is to be performed in real time.

### Limitations

This study has certain limitations. The sample used here was obtained from a single US device, and since different US manufacturers use different processing techniques that influence the final image, these approaches need to be tested on a wider sample in the future. The sample size used here (20 cases) is a very low number. The chi-squared test revealed statistical significance by showing that there is a variance between physicians and the DSS (*p* < 0.05), and since this was meant to be a pilot study, this number was deemed acceptable but should and will be increased in future work. The algorithms developed herein still do not provide a TIRADS classification. This would be the natural next step in the development process along with further testing. In doing this, the algorithms can be then further trained to classify the nodules in more than just absolutes (benign or malignant). Furthermore, the technical solutions developed here as part of the DSS were all independently tested. They need to be integrated into one solution together with the TIRADS classification so that seamless installment and use in the routine workflow can be assessed. Through this integration, future studies employing a larger sample size, larger expert cohort, and a cross-over study design are planned.

## 5. Conclusions

Different physician experience levels influence nodule detection and region estimation, as seen through the classification match percentages and variance percentages. This difference in observations can be minimized by the use of the DSS and in turn reduce observer variability. Additionally, the use of the DSS could also help physicians with less experience in image-based diagnosis to rapidly increase their experience levels by providing them with constant feedback and aid throughout the diagnostic process.

## Figures and Tables

**Table 1:** Classification match percentages between all physicians and DSS, and individual physicians and DSS.

Instance	Classification Match Percentage (%)
All physicians vs. DSS	19.2
Physician 1 vs. DSS	57.6
Physician 2 vs. DSS	42.3
Physician 3 vs. DSS	46.1

**Table 2:** Comparison of observations between three physicians and DSS in estimating the cystic region percentages in US thyroid nodules.

Nodule Number	Physician 1 Cystic (%)	Physician 2 Cystic (%)	Physician 3 Cystic (%)	DSS Cystic (%)
1	10	10	5	8
2	15	25	5	16
3	15	5	5	14
4	50	50	20	27
5	90	95	0	84
6	0	20	70	22
7	75	70	70	20
8	10	10	10	9
9	45	50	20	1
10	5	5	0	0
11	95	80	80	28
12	50	30	20	16
13	30	30	20	3
14	0	0	0	0
15	95	30	90	27
16	15	20	15	5
17	10	50	5	0
18	5	15	0	0
19	1	5	0	0
20	5	20	5	7
21	0	5	0	0
22	10	10	5	3
23	2	15	5	0
24	70	75	50	66
25	70	10	60	8
26	1	5	0	0

**Table 3:** Variance percentages between each physician and DSS.

Nodule Number	VP Physician 1 and DSS (Cystic %)	VP Physician 2 and DSS (Cystic %)	VP Physician 3 and DSS (Cystic %)
1	20	20	−60
2	−6.6	36	−220
3	6.6	−180	−180
4	46	46	−35
5	6.6	11.5	NA
6	NA	−10	68.57
7	73.3	71.4	71.42
8	10	10	10
9	97.7	98	95
10	100	100	NA
11	70.5	65	65
12	68	46.6	20
13	90	90	85
14	0	0	0
15	71.5	10	70
16	66.6	75	66.6
17	100	100	100
18	100	100	NA
19	100	100	0
20	−40	65	−40
21	0	100	0
22	70	70	40
23	100	100	100
24	5.7	12	−32
25	88.5	20	86.6
26	100	100	0

## Data Availability

Not applicable.
